# Golf Swing-Induced Pacemaker Atrial Noise and Extraction: A Case Report and Literature Review

**DOI:** 10.7759/cureus.63409

**Published:** 2024-06-28

**Authors:** Ghassan Akkad, Leo Meller, Matthew Allen, Katherine Wilson, Kenneth Vitale

**Affiliations:** 1 Department of Orthopedic Surgery, Division of Sports Medicine, University of California San Diego School of Medicine, La Jolla, USA

**Keywords:** interdisciplinary cardiac care, neuromusculoskeletal evaluation, repetitive motion icd failure, sport-specific cardiac assessment, cardiac device biomechanical impact, atrial lead noise, golf swing pacemaker interference

## Abstract

Implantable medical devices, such as pacemakers, have significantly improved the quality of life for patients with cardiac conditions, allowing them to maintain active lifestyles. Nonetheless, these devices can present unique challenges when interacting with the wearer's physical activities, potentially leading to unforeseen complications. Here, we present a case of an 81-year-old male golfer, with a history of atrial fibrillation, congestive heart failure, and sick sinus syndrome, who experienced atrial lead noise from his pacemaker, exclusively triggered by his golf swing. This incident, which led to multiple interventions including lead extraction, reimplantation, and eventually a switch to a unipolar lead configuration, represents the first documented case of its kind. It underscores the intricate relationship between the biomechanical forces of certain sports and the functionality of implanted cardiac devices. Through detailed electrophysiology testing, this case demonstrates how specific movements inherent to the patient's golf swing could induce micro-damage to the pacemaker leads, causing noise and malfunction. The findings from this case emphasize the need for healthcare providers to perform sport-specific biomechanical evaluations and create tailored rehabilitation strategies that consider the unique physical demands placed on patients with implanted devices. This approach is important not only for diagnosing and managing similar cases but also for advancing our understanding of how to best support the active lifestyles of patients with implanted cardiac devices, ensuring their safety and longevity.

## Introduction

Participation in the sport of golf in the United States is the highest it has ever been. In 2022, one in seven Americans reported playing golf [1]. Given its low-impact nature, golf serves as an accessible sport for individuals of advanced age to maintain physical activity and engagement. The diversity of participants, including individuals who may not participate in other more demanding sports, lends itself to a variety of injuries. While intense cardiac exertion may not come immediately to mind when considering golf, one study did observe cardiac-related injuries, most commonly in golfers 55 years and older, including palpitations, arrhythmias, and myocardial infarctions [2]. The most common golf-related injuries are trauma from being struck by a golf club or ball [2]. The majority of injuries in golfers are observed among two age groups: those aged 7-17 and individuals over 55 years old. Notably, the latter group experiences a fivefold increase in hospitalization rates due to their injuries [2].

Since their clinical implementation in 1980, implantable cardioverter defibrillators (ICDs) have improved the survival and quality of life of patients at risk of sudden cardiac death [3]. These devices have enabled many to maintain relatively normal lives, including the continuance of physical activity. While the indications for ICDs in athletes are generally the same as in the general population, the athletic heart faces unique stresses; thus, strict guidelines were developed during the 36th Bethesda Conference regarding physical activity levels in patients with ICDs [4]. Contact sports are universally discouraged among athletes with ICDs due to the high likelihood of damage to the device [5].

In contrast, non-contact, low-impact sports are thought to be low-risk and generally accepted for athletes using ICDs. Golf, while considered a low-risk sport by current guidelines in North America, has been reported in a survey to facilitate ICD malfunction [6]. Thus, further consideration of the guidelines for ICDs in athletes is warranted to ensure these athletes are kept safe, while not unnecessarily restricted from low-risk activities. Herein, we present a case of an 81-year-old male golfer with a pacemaker, whose golf swing caused lead noise, necessitating medical intervention.

## Case presentation

An 81-year-old male golfer with a history of atrial fibrillation, chronic obstructive pulmonary disease, congestive heart failure with reduced ejection fraction, and sick sinus syndrome presented to a sports medicine clinic with knee pain associated with golfing. Notably, the patient also reported atrial lead noise associated with golf swing. The patient underwent initial dual-chamber Abbott pacemaker implantation in 2009 for sick sinus syndrome. However, he underwent lead extraction and reimplantation in 2019 due to atrial lead noise during specific arm movements. The patient underwent cardiac electrophysiology testing and tried to reproduce this atrial lead noise with certain arm positions such as raising overhead, pulling, pushing, and resistance exercises. However, it was not reproducible until he simulated a golf swing involving his left arm crossing over the right with his hands adjoined, mimicking a swing.

**Figure 1:**
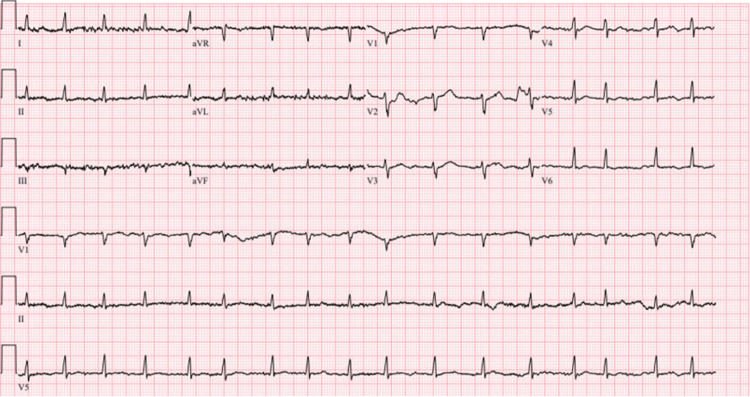
Pre-procedure ECG indicating atrial fibrillation with rapid ventricular response, low-voltage QRS, and nonspecific T wave abnormality before lead extraction and reimplantation. Ventricular rate: 101 bpm, QRS: 78 ms, QT/QTcB: 358/464 ms (ECG obtained on 11/12/2023). ECG settings: speed 25 mm/s; amplitude 10 mm/mV; filter 150 Hz.

In May 2023, the right ventricular lead was switched to unipolar due to a right ventricular impedance drop. Figure 1 represents the initial ECG obtained in November 2023, which demonstrates atrial fibrillation with rapid ventricular response, indicative of an irregular and often rapid heart rate that can lead to poor blood flow. Accompanying low-voltage QRS suggests potential pulmonary disease, pericardial effusion, or a normal variant, warranting further clinical evaluation. Nonspecific T wave abnormalities are noted, contributing to the classification of this ECG as abnormal. This ECG underlines the complex cardiac presentation necessitating careful assessment and intervention. Ongoing pacemaker lead irritation mandated imminent lead replacement, and in December 2023, the patient underwent successful right ventricular lead extraction and reimplant.

Following the procedure, cardiology recommendations allowed for the cautious resumption of activities with restrictions on arm movements to facilitate recovery; the patient was advised to avoid sports with excessive arm swinging, including golf, for six weeks. The patient eventually returned to golf, indicating a positive trend in clinical management and lifestyle integration post-treatment.

**Figure 2:**
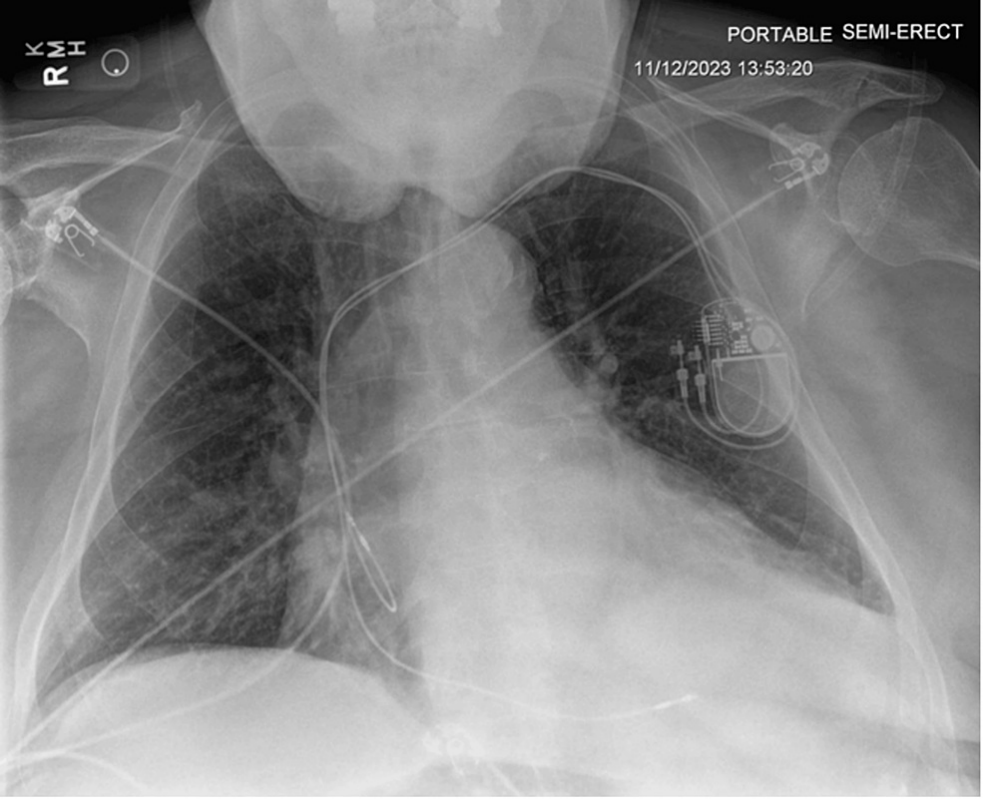
Pre-procedure posteroanterior chest X-ray showing pacemaker position before lead extraction (X-ray obtained on 11/12/2023)

Figure 2 represents the X-ray acquired before lead extraction and reimplantation, demonstrating a well-positioned dual-chamber pacemaker despite clinical symptoms necessitating intervention. The cardiac silhouette is enlarged; no significant pulmonary or pleural abnormalities are noted.

**Figure 3:**
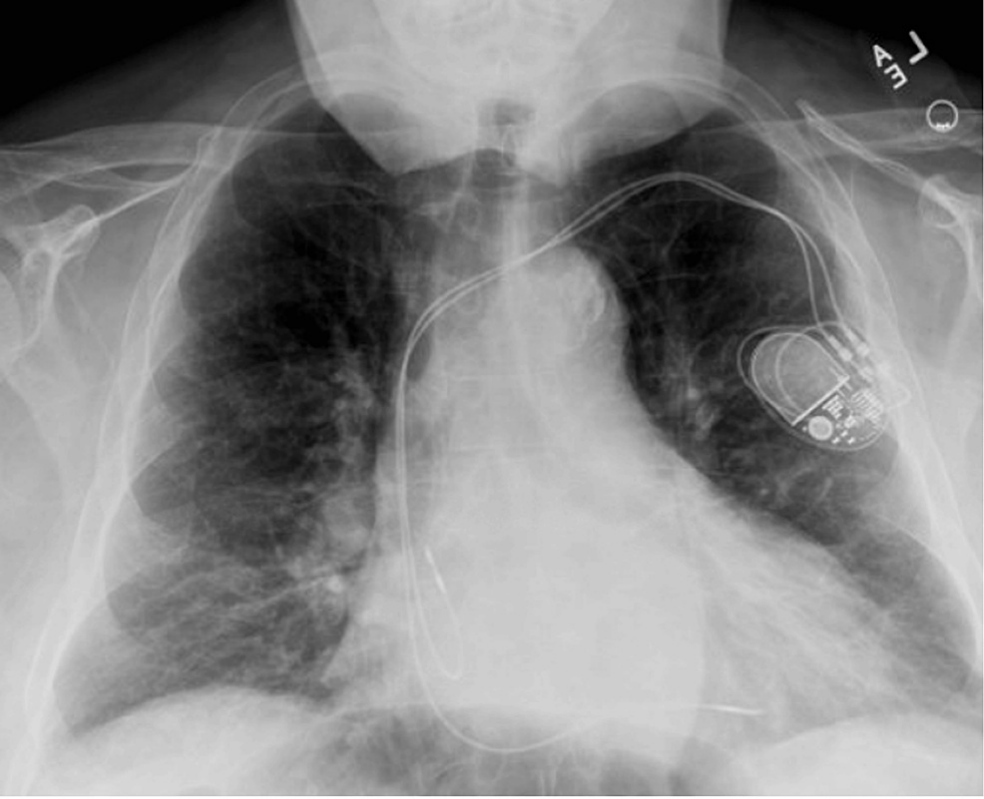
Post-procedure posteroanterior chest X-ray following right ventricular lead reimplantation (X-ray obtained on 12/5/2023)

Figure 3 represents the X-ray acquired after lead extraction and reimplantation, confirming successful right ventricular lead repositioning with adequate pacemaker placement. Mild cardiomegaly is noted; lungs remain clear, indicating no procedural complications.

This sequence of X-rays highlights the critical importance of integrating clinical evaluations with imaging to guide patient care, especially in patients with ICDs who are active in sports. Our case demonstrates that clinical symptoms, such as lead noise, may only manifest during certain sport-specific physical activities. This finding suggests that standard imaging while appearing unremarkable might not capture the mechanical stress exerted on pacemaker leads during dynamic motions, such as a golf swing. It underscores the necessity of activity-specific assessments and detailed patient history in managing patients with ICDs (Table 1).

**Table 1:** Timeline of key medical events

Date	Event
2009	Initial dual-chamber Abbott pacemaker implantation for sick sinus syndrome
2019	Lead extraction and reimplantation due to atrial lead noise during specific arm movements
May 2023	Right ventricular lead switched to unipolar due to a right ventricular impedance drop
November 2023	ECG obtained, demonstrating atrial fibrillation with rapid ventricular response
December 2023	Successful right ventricular lead extraction and reimplantation

## Discussion

This case report documents a unique instance where repetitive golf swings caused atrial noise in a patient with an ICD, necessitating lead intervention. This case highlights the complex interplay between physical activity and the functionality of implanted medical devices. It highlights the need for a deeper understanding of the biomechanical forces at work in sport-specific activities and their potential impacts on such devices. The mechanism of injury, in this case, is likely attributed to cumulative microdamage caused by repetitive golf swings, compromising the pacemaker lead integrity. Each swing involves significant rotational forces and repetitive arm movements that can exert undue strain on the pacemaker leads connecting the implanted device to the heart. These repeated mechanical stresses likely caused degradation of the lead insulation over time. When paired with the unique biomechanical forces and high-velocity movements involved in a golf swing, this degradation may lead to temporary deformation of the leads, intermittently disrupting the electrical signal and resulting in lead noise. Scapular imbalances can further exacerbate this issue by altering arm movement biomechanics and increasing stress on pacemaker leads. Addressing these imbalances through targeted scapular strengthening and mobility training could help mitigate mechanical stresses. A thorough neuromusculoskeletal examination focusing on sport-specific movements for athletes with ICDs is crucial, potentially identifying and addressing biomechanical imbalances that may lead to complications.

The distinctiveness of this case report can be better appreciated in the context of existing literature on ICDs and sports, which primarily focuses on recommendations for or against participation in various levels of sports for individuals with pacemakers [7-9]. Current recommendations classify different activities in terms of static (I - low, II - moderate, III - high) and dynamic (A - low, B - moderate, C - high) intensity using such ratings to advise specific patients on participation or non-participation in certain sports [10]. These guidelines are largely based on expert consensus. Robust safety data on athletic participation by specific sub-populations with ICDs participating in specific sports is lacking [5,11]. Such a gap may result in individuals being inappropriately prevented from participating in sports that could be beneficial and of low risk for them or could result in others not fully appreciating the risks associated with their participation in a particular sport. Thorough guidelines that support an individually tailored approach are needed [9].

Adverse events caused by ICDs during sports are most commonly electrical shocks with other significant adverse events being extremely rare [12]. Among adverse events known to arise in athletes with ICDs, lead fracture or dislodgement due to repetitive motion sports is the most common [6]. Existing case reports on athletic participation by individuals with ICDs largely focus on young athletes participating in vigorous sports. This focus holds true even for literature focusing on the specific issue of lead fracture or damage with some studies specifically excluding golf from the analysis [13,14].

This narrowed focus may inadvertently obscure insights into the prevalence and mechanisms of injury for older individuals with ICDs engaging in lower-intensity sports, potentially resulting in underdiagnosis or delayed diagnosis. Furthermore, golf is classified as an IA (low static intensity, low dynamic intensity) activity, meaning that it is recommended as appropriate for individuals with ICDs [10]. Our case study illustrates that even those engaging in low-intensity activities need to be monitored and educated on the associated risks of athletic participation with ICDs. Guidelines that focus only on dynamic or static intensity may fail to appreciate unique aspects of lower-intensity sports such as golf which can lead to ICD complications. However, it is important to note that while the patient in our case experienced lead noise causing multiple rounds of lead extraction and reimplantation, there were no reported safety events. Individuals with ICDs should not be automatically precluded from participating in low-intensity activities. Rather, patients and clinicians should be educated on the possible effects of repetitive motion activities on ICD leads to facilitate informed consent and timely diagnosis and treatment. The decision to participate in low-intensity activities should be based on individual circumstances and clinical judgment.

## Conclusions

Addressing complications related to ICDs in athletes requires an integrated and comprehensive approach. This strategy encompasses a thorough neuromusculoskeletal examination, with a particular focus on sport-specific movements in sports with repetitive upper-body movements, such as golf. The aim of the exam is to identify biomechanical imbalances or asymmetries, particularly in the shoulder and upper extremity areas, that could exert undue strain on an implanted device such as a pacemaker. Assessment and strengthening of the scapulohumeral rhythm are critical components of this approach. Scapular mobility and stability are critical for the safe and effective performance of upper extremity movements. Rehabilitation programs may be prescribed to reduce the risk of device-related injuries by strengthening the scapular stabilizers, as well as the upper back and shoulder muscles. These targeted exercises ensure that athletes, particularly golfers, can participate in their chosen pastime without putting unnecessary strain on their implanted devices. Ultimately, collaboration between physiatrists, sports medicine specialists, and cardiologists is critical to creating a comprehensive care plan that balances the benefits of physical activity with the safety and functionality of ICDs.
